# Short-Term Outcomes of Switching to Ranibizumab in Polypoidal Choroidal Vasculopathy Resistant to Aflibercept Therapy

**DOI:** 10.3390/jcm10245739

**Published:** 2021-12-08

**Authors:** Young-Joon Jeon, Jae-Hui Kim, Jong-Woo Kim, Chul-Gu Kim

**Affiliations:** Department of Ophthalmology, Kim’s Eye Hospital, Seoul 150-034, Korea; yjipnida@kimeye.com (Y.-J.J.); kjwood@kimeye.com (J.-W.K.); chulgukim@kimeye.com (C.-G.K.)

**Keywords:** polypoidal choroidal vasculopathy, aflibercept, ranibizumab, switching, resistant, refractory

## Abstract

Background: To evaluate the short-term outcomes of switching to ranibizumab in aflibercept-resistant polypoidal choroidal vasculopathy (PCV). Methods: This retrospective study included 18 eyes diagnosed with aflibercept-resistant PCV. All patients were treated with two to four consecutive ranibizumab injections at 4–5-week intervals. The best-corrected visual acuity (BCVA), and central retinal thickness (CRT) values before and after switching to ranibizumab were compared. The proportion of eyes showing ≥100 µm decrease in retinal thickness and/or complete resolution of fluid after switching was identified. Results: The mean number of aflibercept injections before switching was 5.7 ± 3.3. After switching, a mean of 2.8 ± 0.6 consecutive ranibizumab injections was performed. The mean logarithm of minimal angle of resolution (logMAR) BCVA was 0.41 ± 0.26 (Snellen equivalents = 20/51) before switching, and 0.40 ± 0.30 (20/50) after switching (*p* = 0.574). The mean CRT was 422.2 ± 152.4 µm before switching, and 400.7 ± 182.0 µm after switching (*p* = 0.236). A decrease in CRT of ≥100 µm, and/or complete resolution of fluid was noted in three eyes (16.7%). Conclusions: Switching to ranibizumab in aflibercept-resistant polypoidal choroidal vasculopathy was not effective in most patients, suggesting the need for further investigation to seek more effective treatment options for this condition.

## 1. Introduction

Polypoidal choroidal vasculopathy (PCV) is a distinct type of choroidal neovascularization (CNV) that is characterized by polypoidal lesions and branching vascular networks [1]. Previously, photodynamic therapy (PDT) was used as the primary treatment option for this condition. However, anti-vascular endothelial growth factor (VEGF) therapy has widely replaced PDT as an effective first-line treatment for PCV [2,3].

Ranibizumab and aflibercept are widely used as effective anti-VEGF drugs to treat PCV [2]. However, there are some differences in the characteristics and efficacy of these two drugs. Ranibizumab inhibits VEGF-A, whereas aflibercept inhibits VEGF-A, VEGF-B, and placental growth factor [4]. In addition, aflibercept demonstrates a longer VEGF suppression time than ranibizumab [5]. In PCV, eyes treated with aflibercept usually show a greater reduction in macular thickness, and higher polyp regression rate than those treated with ranibizumab [6,7].

It is well known that some PCVs are resistant to ranibizumab therapy [8]. In this case, switching to aflibercept could be a useful treatment option leading to anatomical and functional improvement [9]. However, to date, a paucity of knowledge exists regarding the efficacy of switching to ranibizumab in aflibercept-resistant PCV. Recently, Hara et al. investigated tachyphylaxis during aflibercept therapy in neovascular age-related macular degeneration (AMD) [10]. Although PCV cases were included in that report, ranibizumab therapy was only performed in selected cases [10]. The efficacy of switching to ranibizumab in aflibercept-resistant PCV remains to be elucidated.

PCV is present in various racial and ethnic populations [2,11], with an especially higher prevalence in Asian populations [2]. Since aflibercept is a widely used anti-VEGF treatment for PCV [12,13], establishing an appropriate treatment strategy for aflibercept-resistant PCV is of great value. The current study addresses the question of whether switching to ranibizumab could be a useful alternative in aflibercept-resistant PCV. Hence, the short-term anatomical and functional outcomes of switching to ranibizumab in aflibercept-resistant PCV were evaluated.

## 2. Materials and Methods

This retrospective observational study was conducted at a single center (Kim’s Eye Hospital, Seoul, Korea). The study was approved by the Institutional Review Board of Kim’s Eye Hospital and was conducted in accordance with the tenets of the Declaration of Helsinki. Due to the retrospective nature of this study, the need for an informed consent was waived (Kim’s Eye Hospital IRB, Seoul, Korea).

### 2.1. Patients

This study included patients diagnosed with PCV between January 2014 and December 2019. In all the included patients, fluorescein angiography and indocyanine-green angiography (ICGA) was performed at diagnosis using a Spectralis HRA+OCT^®^ device. The PCV was diagnosed when polypoidal lesions with or without branching vascular networks were noted on ICGA images. Initially, a single experienced examiner (J.-H.K.) analyzed ICGA images to diagnose PCV. Later, another examiner (Y.-J.J.) analyzed the images from cases diagnosed as PCV to confirm diagnostic accuracy. Additional inclusion criteria were as follows: (1) initially treated with aflibercept (2.0 mg/0.05 mL of Eylea^®^; Regeneron, Tarrytown, NY, USA), (2) anti-VEGF drug was switched to ranibizumab (0.5 mg/0.05 mL of Lucentis^®^; Genentech Inc., San Francisco, CA, USA) during the follow-up due to limited response to aflibercept. The exclusion criteria were as follows: (1) history of vitreoretinal surgery, PDT, or bevacizumab treatment; and (2) other vitreoretinal disorders that may influence macular microstructure and visual function.

### 2.2. Treatment

Patients were initially treated with three loading injections of intravitreal aflibercept. Re-treatment was performed as needed. In selected cases, the treatment regimen was changed to treat-and-extend (TAE). There was no strict guideline to switching treatment regimen. The regimen was changed when the treating physician determined that more effective treatment was required to preserve vision. The anti-VEGF agent was switched to ranibizumab when the treating physician determined that the response to aflibercept was limited. No strict guidelines have been established for determining aflibercept resistance or switching of anti-VEGF agents. In general, aflibercept resistance was determined when no improvement (≥100 µm) or worsening of the central retinal thickness (CRT) was detected within 4–6 weeks of 2–3 consecutive aflibercept injections. After switching, two to four consecutive ranibizumab injections were administered at 4–5-week intervals. The patient was scheduled to visit the hospital 4–5 weeks after ranibizumab injections to evaluate outcomes after switching. When using aflibercept, the minimum interval between injections was set as two months, except for loading injections. When using ranibizumab, the minimum interval between injections was set as one month. All the optical coherence tomography (OCT) examinations were performed using a Spectralis HRA+OCT^®^ (Heidelberg engineering, Heidelberg, Germany) device. Horizontal and vertical cross-hair scans centered at the center of the fovea were performed. Raster scans of macular area with 31 scanning lines, were also performed to obtain CRT value.

### 2.3. Outcome Measures

The following data were collected: patients’ age and sex, lens status, period between the diagnosis and switching, and number of aflibercept injections before switching. The best-corrected visual acuity (BCVA), presence of fluid on OCT, and CRT before and after switching were evaluated, and the number of consecutive ranibizumab injections was recorded. Before and after switching to ranibizumab, manifest refraction was performed to accurately detect the refractive error. BCVA was measured wearing trial glasses based on manifest refraction. The BCVAs were measured using a decimal visual acuity chart, then converted to logarithm of the minimal angle of resolution (logMAR) values for analysis.

Eyes were divided into two groups according to the decrease in CRT after switching: eyes showing ≥50 µm decrease in CRT were included in the responder group, whereas the remaining eyes were included in the non-responder group. The following parameters were compared between the two groups: patients’ age and sex, diabetes mellitus, hypertension, lens status, period between diagnosis and switching, number of aflibercept injections before switching, BCVA and CRT at switching, and number of consecutive ranibizumab injections.

To elucidate difference in imaging features between the responder group and the non-responder group, the following baseline imaging features were compared between the two groups: choroidal vascular hyperpermeability, presence of polyp clusters, greater linear dimension of the lesion, largest polyp diameter, presence of pigment epithelial detachment (PED), and choroidal thickness (≥300 µm vs. <300 µm).

The following parameters were additionally compared between patients treated with as-needed regimen before switching to ranibizumab (as-needed group) and those in which treatment regimen was changed from as-needed to TAE before switching to ranibizumab (TAE group): duration between the diagnosis and the switching, number of anti-VEGF injections before switching to ranibizumab, age, sex, choroidal vascular hyperpermeability, presence of polyp clusters, greater linear dimension of the lesion, largest polyp diameter, proportion of patients with choroidal thickness ≥300 µm, change in CRT after the switching, proportion of patients showing ≥50 µm decrease in CRT after switching.

### 2.4. Statistical Analyses

The data are presented as mean ± standard deviation or as a number (percentage) wherever applicable. Statistical analyses were performed using a commercially available software package (SPSS, version 12.0; IBM Corporation, Armonk, NY, USA). The Shapiro–Wilk test was used to identify the normal distribution of the data. Comparisons before and after switching were performed using the Wilcoxon signed-rank test. Comparisons between responder and non-responder groups were performed using the Mann–Whitney *U* test or Fisher’s exact test. Comparisons between the TAE group and the as-needed group were performed using the Mann–Whitney *U* or Fisher’s exact test. A *p*-value < 0.05 was considered statistically significant.

## 3. Results

A total of 18 eyes from 18 patients (13 men and 5 women) were included in the analysis (Table 1). Since none of 18 patients underwent switching to ranibizumab in both eyes, only one eye in each patient was included. The mean age was 65.8 ± 6.9 years. The period between the initial diagnosis and switching to ranibizumab was 11.7 ± 9.1 months. During this period, 5.7 ± 3.3 aflibercept injections were performed. After switching, 2.8 ± 0.6 consecutive ranibizumab injections were performed. In eight patients, treatment response to initial loading injections of aflibercept was limited, and switching to ranibizumab was performed immediately after the loading injections. In the remaining 10 eyes, treatment response to initial loading injections of aflibercept was satisfactory, but a limited response was noted during the follow-up period.

**Figure 1:**
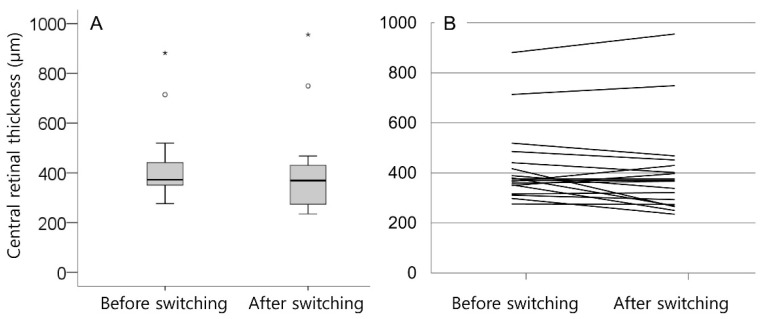
Box plot (**A**) and line graph (**B**) show distribution of central retinal thickness before and after switching to ranibizumab. Line graph (**B**) shows changes in central retinal thickness in all 18 patients separately. Circles and asterisks (**A**) indicate outliers.

**Figure 2:**
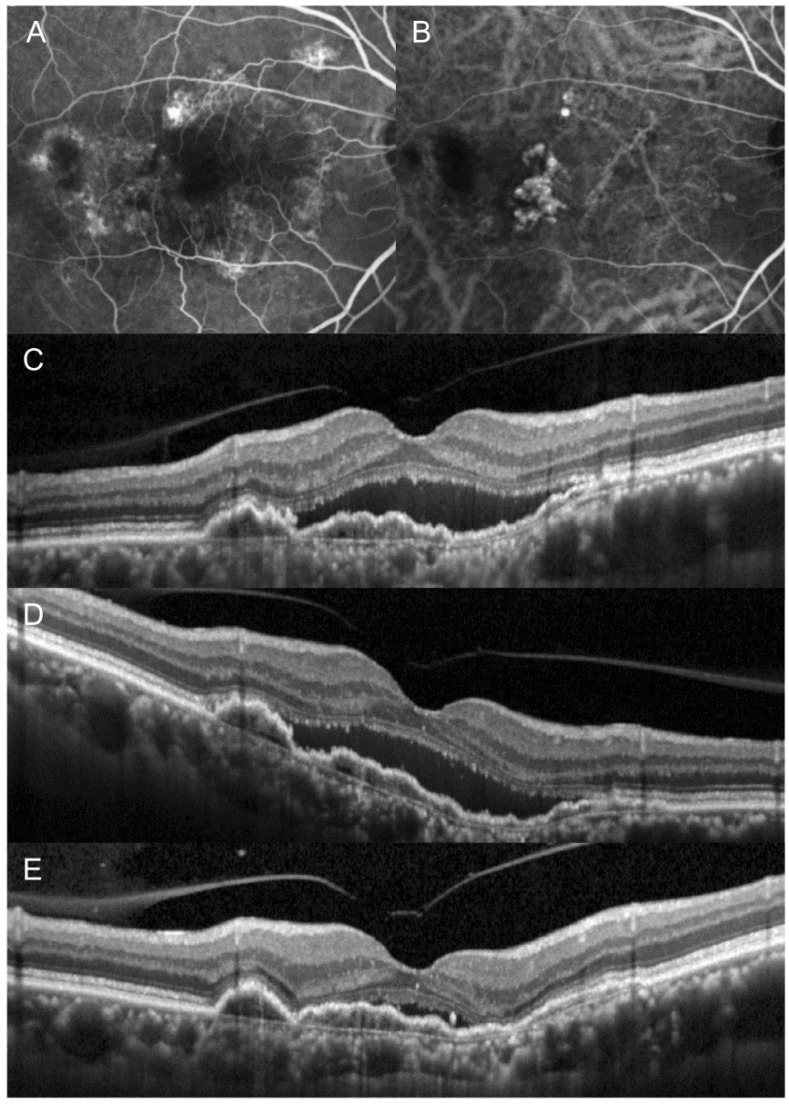
The clinical course of an eye in which fluid was markedly decreased after switching from aflibercept to ranibizumab. At initial visit (**A**–**C**), the patient was diagnosed with polypoidal choroidal vasculopathy. After three loading injections of aflibercept (**D**), subretinal fluid remained stationary and the anti-vascular endothelial growth factor drug was switched to ranibizumab. After three additional ranibizumab injections (**E**), marked resolution of subretinal fluid was noted. (**A**) = fluorescein angiography, (**B**) = indocyanine-green angiography, (**C**–**E**) = optical coherence tomography.

**Figure 3:**
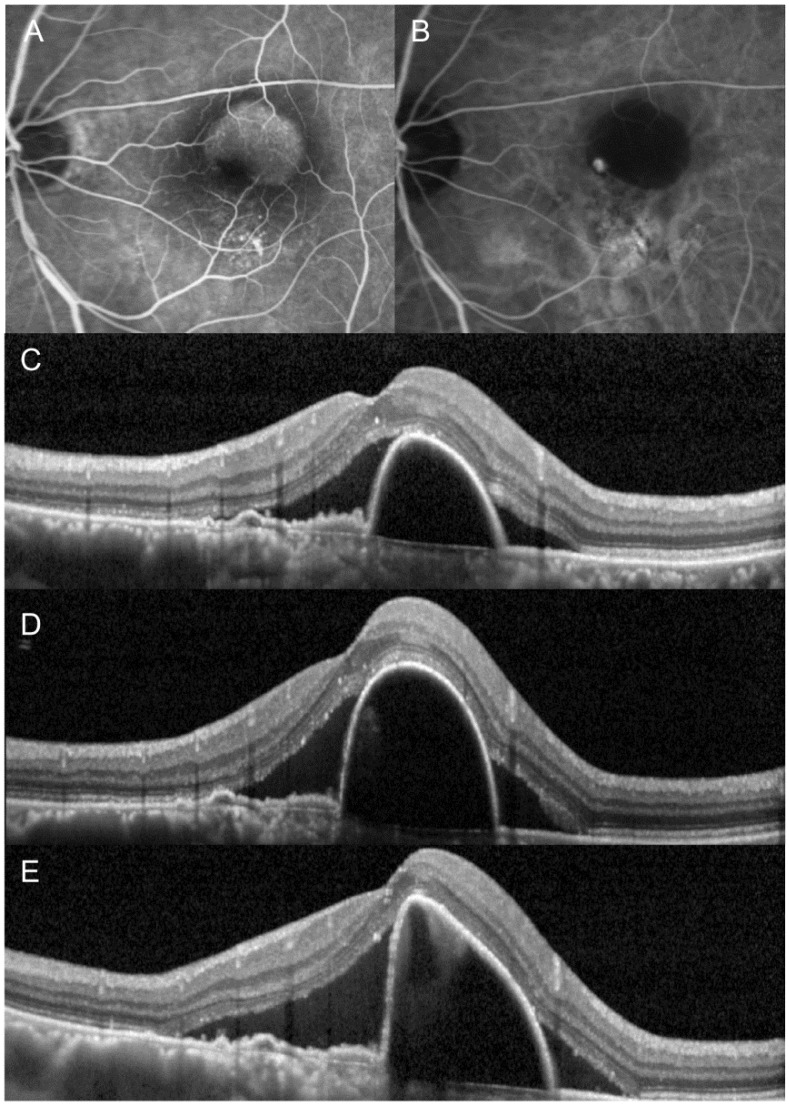
The clinical course of an eye in which fluid did not decrease after switching from aflibercept to ranibizumab. At initial visit (**A**–**C**), the patient was diagnosed with polypoidal choroidal vasculopathy. After three loading injections of aflibercept (**D**), subretinal fluid remained stationary with an increase in the height of pigment epithelial detachment. The anti-vascular endothelial growth factor drug was switched to ranibizumab. However, subretinal fluid increased further after three additional ranibizumab injections (**E**). (**A**) = fluorescein angiography, (**B**) = indocyanine-green angiography, (**C**–**E**) = optical coherence tomography.

Table 2 summarizes the changes in the BCVA and CRT after switching. The mean logMAR BCVA was 0.41 ± 0.26 (Snellen equivalents = 20/51) before switching, and 0.40 ± 0.30 (20/50) after switching. There was no difference in the BCVA before and after switching (*p* = 0.574). After switching, BCVA improved in six eyes (33.3%), decreased in four eyes (22.2%), and remained stationary in eight eyes (44.4%). However, improvement of ≥2 lines in BCVA was not noted in any of the eyes. The mean CRT was 422.2 ± 152.4 µm before switching, and 400.7 ± 182.0 µm after switching (Figure 1). There was no difference in CRT before and after switching (*p* = 0.236). After switching, a decrease in CRT of >100 µm was noted in three eyes (16.7%), and of 50–100 µm was noted in three eyes (16.7%). An increase between 50 and 100 µm CRT was noted in two eyes (11.1%). The remaining eight eyes (55.6%) maintained a stable CRT. Figure 2 and Figure 3 show eyes with and without a decrease in SRF after switching, respectively.

Before switching, SRF was noted in all 18 eyes, and IRF was absent in all eyes. After switching, SRF completely resolved in two eyes (11.1%). However, SRF was still observed in the remaining 16 eyes (88.9%). In the comparison between the responder and non-responder groups (Table 3), there was no difference in the age (*p* = 0.213), sex (*p* = 1.000), diabetes mellitus (*p* = 1.000), hypertension (*p* = 0.321), lens status (*p* = 0.615), duration between diagnosis and switching (*p* = 0.964), number of aflibercept injections before switching (*p* = 0.616), BCVA (*p* = 0.964), CRT (*p* = 0.820), and number of consecutive ranibizumab injections (*p* = 0.750).

There was no difference in choroidal vascular hyperpermeability (*p* = 0.245), presence of polyp clusters (*p* = 0.569), greater linear dimension of the lesion (*p* = 0.051), largest polyp diameter (*p* = 0.291), presence of PED (*p* = 0.638), and choroidal thickness (≥300 µm vs. <300 µm) (*p* = 0.638).

Among 18 patients, 7 were included in the TAE group and 11 were included in the as-needed group. The number of anti-VEGF was significantly greater in the TAE group (mean 9.0 ± 2.6 injections) than in the as-needed group (mean 3.5 ± 1.2 injections) (*p* < 0.001). In addition, the duration between the diagnosis and the switching was significantly greater in the TAE group (mean 18.7 ± 6.7 months) than in the as-needed group (mean 7.3 ± 7.6 months) (*p* = 0.003). Other parameters, including age (*p* = 0.724), sex (*p* = 0.326), choroidal vascular hyperpermeability (*p* = 0.245), presence of polyp clusters (*p* = 0.623), greater linear dimension of the lesion (*p* = 0.375), largest polyp diameter (*p* = 0.065), proportion of patients with choroidal thickness ≥300 µm (*p* = 0.630), change in CRT after the switching (*p* = 0.724), proportion of patients showing ≥50 µm decrease in CRT after the switching (*p* = 1.000), were not different between the two groups.

## 4. Discussion

None of the previous studies have focused on the outcomes of switching to ranibizumab in aflibercept-resistant PCV. Hara et al. [10] evaluated treatment outcomes in 28 eyes that developed aflibercept tachyphylaxis. Among them, 14 patients were diagnosed with PCV. The anti-VEGF agent was switched to ranibizumab in 9 of the 28 eyes. However, the exact number of PCV patients that underwent switching and their outcomes were not separately presented [10]. As far as we are aware, ours is the first study to focus on the outcomes of switching to ranibizumab in aflibercept-resistant PCV.

In the present study, the efficacy of switching to ranibizumab in aflibercept-resistant PCV was limited. The BCVA and CRT after switching were not different from those before switching. A ≥100 µm decrease in CRT and/or complete resolution of fluid was noted in only 16.7% of the patients. In addition, an improvement of ≥2 lines in visual acuity was not observed in any eyes. This result suggests that when PCV is resistant to bimonthly aflibercept injections, switching to ranibizumab may not be beneficial for many patients. Thus, it would be necessary to inform patients that treatment efficacy could be limited if switching to ranibizumab. This raises the question of the most appropriate alternative to ranibizumab for aflibercept-resistant PCV.

PDT was previously used as a first-line treatment for PCV [14], but diminished after the advent of anti-VEGF therapy. However, it is still considered as a useful treatment option for PCV [2]. In a previous study of Takayama et al., PDT in combination with aflibercept was found to yield similar visual and anatomical outcomes as aflibercept monotherapy, while requiring fewer aflibercept injections [15]. However, it is still questionable whether PDT is beneficial in aflibercept-resistant cases because only a few studies have evaluated this issue. In the Aflibercept in Polypoidal Choroidal Vasculopathy (PLANET) study, aflibercept monotherapy was found to be non-inferior to aflibercept with rescue PDT up to 96 weeks [16], suggesting the limited role of PDT in aflibercept-resistant PCV. However, a recent study has demonstrated different results. In the study by Park et al., rescue PDT was beneficial in resolving fluid and improving visual acuity in refractory Type 1 neovascularization, including PCV [17]. This suggests that PDT can be considered as an alternative to aflibercept-resistant PCV.

The second alternative would be a higher or more frequent dose of anti-VEGF injections. In a previous study, 2.0 mg of super-dose ranibizumab led to significant visual and anatomical improvement in neovascular AMD that was resistant to previous 0.5 mg ranibizumab injections [18]. In addition, in the PLANET study, aflibercept injections every four weeks were used as a rescue treatment when the response to aflibercept injections every eight weeks was not adequate [16]. Although the efficacy of higher or more frequent doses of anti-VEGF injections has not been directly evaluated in aflibercept-resistant PCV, results of previous studies suggest that this approach can be considered as a potential treatment option.

The third alternative would be switching to anti-VEGF drugs other than ranibizumab. Recently, a new anti-VEGF drug, brolucizumab, was introduced. In clinical trials, brolucizumab showed superior efficacy to aflibercept in macular thickness reduction [19]. In addition, one recent study presented resolution of SRF after a single brolucizumab injection in an eye which showed limited response to previous aflibercept therapy [20]. Moreover, polypoidal lesions appear to frequently regress after brolucizumab therapy [21]. To date, efficacy with brolucizumab in refractory CNV has not yet been sufficiently investigated. However, considering the results of previous studies [19,20], brolucizumab may be considered as a potential treatment option for aflibercept-resistant PCV.

To date, there are no established guidelines regarding the management of aflibercept-resistant PCV. All the methods described above, including switching to ranibizumab, are merely potential treatment options in which the efficacy has not yet been fully established. Further studies accompanied by expert discussions are needed to elucidate the appropriate treatment strategy for aflibercept-resistant PCV.

In the present study, we attempted to reveal the characteristics related to the decrease in CRT after switching. However, no differences in characteristics were noted between the responder and non-responder groups. In addition, although previous studies have shown that imaging features—including choroidal vascular hyperpermeability, presence of polyp clusters, greater linear dimension of the lesion, largest polyp diameter, and choroidal thickness—can influence the treatment outcomes [22,23,24], there was no significant difference in these features between the responder and the non-responder group. However, we postulate that these results were partially induced by the small sample size in this study.

Serous PED is frequently detected in PCV [25]. Although there has been no report indicating that serous PED is predictive of treatment response for switching anti-VEGF drug, persistent PED can be associated with a high risk of lesion reactivation [26]. In the present study, the incidence of serous PED was slightly higher in the responder group. However, no significant difference in the incidence of serous PED was observed between the responder and non-responder groups. Considering the small sample size, further studies with a larger study population are needed to confirm our findings.

In the present study, the cut-off value was set as 50 µm, as we believe that this value far exceeds the known variability of CRT measurements. A previous study has shown that there can be up to 15 µm of CRT measurement variability when using a Spectralis^®^ device [27]. Accordingly, we postulate that a 3-fold decrease in a CRT of 15 µm indicates reduced thickness, regardless of measurement variability.

Herein, 30.8% of males and 40.0% of females were included in the responder group. A previous study has shown that a greater number of males than females tend to require retreatment for recurrence during the first year of neovascular AMD treatment [28]. To date, however, there was no evidence suggesting any difference in efficacy following switching of anti-VEGF agents between males and females. Hence, it remains unclear why a slightly higher proportion of women responded to ranibizumab. Further studies with a larger study population are required to better understand any gender-related differences in the efficacy of switching of anti-VEGF agents.

The strength of the present study was the focus on the treatment outcomes after switching to ranibizumab in aflibercept-resistant PCV. However, there are limitations to the present study. First, this was a retrospective study based on a small sample size. It is possible that this small sample size influenced results of comparisons between the responder and the non-responder group. Second, there was no strict parameter for determining aflibercept resistance and the switch to ranibizumab. Third, the number of consecutive ranibizumab injections varied between two and four. Fourth, since the minimum prescribed interval between aflibercept injections is two months in South Korea, none of the included eyes received more frequent aflibercept injections. Thus, the present study could not evaluate the outcome in eyes showing a limited response to less than two-month intervals of aflibercept injections. Fifth, the cut-off value to determine the responder (50 μm) was arbitrarily set. Sixth, most image analyses were initially performed by a single examiner. Lastly, the maximum number of consecutive ranibizumab injections was four. Thus, the results of the present study may not be valid when a greater number of consecutive ranibizumab injections is administered.

In summary, we evaluated the short-term efficacy of switching to ranibizumab in aflibercept-resistant PCV. Switching to ranibizumab was not effective in most patients. The limited efficacy of switching to ranibizumab suggests the need for further investigations to seek more effective treatment options for aflibercept-resistant PCV.

## Figures and Tables

**Table 1:** Baseline characteristics of patients (*n* = 18) with aflibercept-resistant polypoidal choroidal vasculopathy included in the trial.

Characteristics	Value
Age (years)	65.8 ± 6.9
Sex (male:female)	13 (72.2%): 5 (27.8%)
Diabetes mellitus	5 (27.8%)
Hypertension	10 (55.6%)
Lens status (phakia:pseudophakia)	13 (72.2%):5 (27.8%)
Duration between the diagnosis and the switching (months)	11.7 ± 9.1
No. of aflibercept injections before the switching	5.7 ± 3.3
Type of fluid when the switching was performed	
Subretinal fluid	18 (100.0%)
Intraretinal fluid	0

Data are presented as mean ± standard deviation or No. (%) when applicable. “Switching” indicates a change from aflibercept to ranibizumab treatment.

**Table 2:** Change in best-corrected visual acuity and central retinal thickness in patients with aflibercept-resistant polypoidal choroidal vasculopathy after switching to ranibizumab.

Change in Best-Corrected Visual Acuity	No. of Eyes (%)
Improved ≥ 2 lines	0
Improved < 2 lines	6 (33.3%)
Stationary	8 (44.4%)
Deteriorated < 2 lines	3 (11.1%)
Deteriorated ≥ 2 lines	1 (5.6%)
Central retinal thickness	
Decreased ≥ 100 µm	3 (16.7%)
Decreased < 100 µm, >50 µm	3 (16.7%)
Stationary	10 (55.6%)
Increased < 100 µm, >50 µm	2 (11.1%)
Increased ≥ 100 µm	0

Data are presented as mean ± standard deviation or No. (%) when applicable.

**Table 3:** Comparison of the characteristics of the responder group and the non-responder group in patients with aflibercept-resistant polypoidal choroidal vasculopathy after switching from aflibercept to ranibizumab treatment.

Characteristics	Responder Group (*n* = 6)	Non-Responder Group (*n* = 12)	*p*-Value
Age (years)	63.0 ± 7.6	67.2 ± 6.3	0.213 *
Sex			1.000 ^†^
Male	4 (66.7%)	9 (75.0%)	
Female	2 (33.3%)	3 (25.0%)	
Diabetes mellitus	2 (33.3%)	3 (25.0%)	1.000 ^†^
Hypertension	2 (33.3%)	8 (66.7%)	0.321 ^†^
Lens status			0.615 ^†^
Phakia	5 (83.3%)	8 (66.7%)	
Pseudophakia	1 (16.7%)	4 (33.3%)	
Duration between the diagnosis and the switching (months)	12.2 ± 10.4	11.5 ± 8.9	0.964 *
No. of aflibercept injections before the switching	6.5 ± 4.7	5.3 ± 2.5	0.616 *
Best-corrected visual acuity (logMAR)	0.39 ± 0.23 (20/49 ^‡^)	0.41 ± 0.29 (20/51 ^‡^)	0.964 *
Central retinal thickness (µm)	392.5 ± 73.9	437.0 ± 180.8	0.820 *
No. of consecutive ranibizumab injections	2.7 ± 5.2	2.8 ± 7.2	0.750 *

Data are presented as mean ± standard deviation or No. (%) when applicable. logMAR = logarithm of minimal angle of resolution; *: Mann–Whitney *U* test; ^†^: Fisher’s exact test; ^‡^: Snellen equivalents.

## Data Availability

The datasets of the present study are available from the corresponding author on reasonable request.
